# Supplementary material for: Polyketide synthase gene clusters in the ericoid mycorrhizal fungus Oidiodendron maius: genome-wide prediction, expression profiling, and functional characterization of knockout mutants
Source: Front Plant Sci. 2026 Sep 16;17:1924430. doi: 10.3389/fpls.2026.1924430 (PMC13623903; doi:10.3389/fpls.2026.1924430)
Supplement: Supplementary file 1 [file SupplementaryFile1.zip › Supplementary Materials/Supplementary Folder 1.zip!/Oidma1_AssemblyScaffolds_Repeatmasked/knownclusterblast/cluster8/jgi.p_Oidma1_23737_mibig_hits.html]

| MIBiG Protein | Description | MIBiG Cluster | MiBiG Product | % ID | % Coverage | BLAST Score | E-value |
| --- | --- | --- | --- | --- | --- | --- | --- |
| AGN71610.1 | putative\_quinone-oxidoreductase-like\_protein | BGC0000027 | Azaphilone\_biosynthetic\_gene\_cluster | 44 | 110.029498525 | 285 | 1e-76 |
| EHA28239.1 | hypothetical\_protein | BGC0001143 | Azanigerone\_biosynthetic\_gene\_cluster | 44 | 102.654867257 | 249 | 4.7e-66 |
| EAA65598.1 | hypothetical\_protein | BGC0000022 | Asperfuranone\_biosynthetic\_gene\_cluster | 39 | 112.684365782 | 223 | 2.8e-58 |
| EAA59568.1 | hypothetical\_protein | BGC0000057 | F9775\_biosynthetic\_gene\_cluster | 36 | 104.424778761 | 212 | 8.3e-55 |
| CBF73513.1 | conserved\_hypothetical\_protein | BGC0001121 | Orsellinic\_acid\_biosynthetic\_gene\_cluster | 36 | 104.424778761 | 212 | 8.3e-55 |
| AGO65991.1 | putative\_enoylreductase-like\_protein | BGC0000992 | Fusaridione\_A\_biosynthetic\_gene\_cluster | 32 | 79.3510324484 | 123 | 3.9e-28 |
| AAX56379.1 | predicted\_zinc-binding\_oxidoreductase | BGC0000127 | Pyoluteorin\_biosynthetic\_gene\_cluster | 30 | 79.3510324484 | 89 | 6.2e-18 |
| AFJ52692.1 | enoyl\_reductase | BGC0001073 | Kosinostatin\_biosynthetic\_gene\_cluster | 34 | 53.982300885 | 70 | 5.1e-12 |
| BAJ16468.1 | polyketide\_synthase | BGC0000058 | FD-891\_biosynthetic\_gene\_cluster | 32 | 55.4572271386 | 68 | 1.5e-11 |
| BAG85026.1 | putative\_polyketide\_synthase | BGC0000086 | Lasalocid\_biosynthetic\_gene\_cluster | 28 | 61.0619469027 | 57 | 2.6e-08 |
| CAQ64686.1 | lasalocid\_modular\_polyketide\_synthase | BGC0000087 | Lasalocid\_biosynthetic\_gene\_cluster | 28 | 61.0619469027 | 57 | 2.6e-08 |
| ACB46197.1 | polyketide\_synthase | BGC0000989 | Epothilone\_biosynthetic\_gene\_cluster | 28 | 52.5073746313 | 50 | 4.2e-06 |
| AAF62885.1 | EpoF | BGC0000991 | Epothilone\_biosynthetic\_gene\_cluster | 28 | 52.5073746313 | 50 | 4.2e-06 |
| ADB12493.1 | EpoF | BGC0000990 | Epothilone\_biosynthetic\_gene\_cluster | 27 | 52.5073746313 | 50 | 5.5e-06 |
